# The Role of Five Facets of Mindfulness in a Mindfulness-Based Psychoeducation Intervention for People With Recent-Onset Psychosis on Mental and Psychosocial Health Outcomes

**DOI:** 10.3389/fpsyt.2020.00177

**Published:** 2020-03-11

**Authors:** Wai Tong Chien, Ka Ming Chow, Yuen Yu Chong, Daniel Bressington, Kai Chow Choi, Carmen Wing Han Chan

**Affiliations:** ^1^The Nethersole School of Nursing, The Chinese University of Hong Kong, Shatin, Hong Kong; ^2^School of Nursing, The Hong Kong Polytechnic University, Kowloon, Hong Kong

**Keywords:** five facets, mindfulness, psychosis, functioning, psychotic symptoms

## Abstract

**Objective:** This study aimed to examine how five facets of mindfulness may be associated with the changes in psychotic patients' health outcomes after participating in a mindfulness-based psychoeducation group (MBPEG) program.

**Methods:** Longitudinal follow-up data from two pragmatic randomized controlled trials of MBPEG for psychotic patients were used for this study. A total of 124 patients who completed the MBPEG program were included in this analysis. Patient outcomes (psychotic symptoms, functioning, insight into illness/treatment, subjective recovery) and five facets of mindfulness were assessed at baseline and six, 12 and 24 months post-intervention. Areas under the curve of individual outcomes in repeated-measures were computed using trapezoidal method, rescaled to the original possible range of the underlying variable and used for correlation and regression analyses.

**Results:** All mean scores of the five facets increased across time and were significantly correlated with the improvements in all patient outcomes (*p-values* ranged from <0.001 to <0.05), except “non-judging” facet and symptom severity. Regression analyses revealed that only “observing” and “acting with awareness” were significantly associated with positive changes across all outcomes (increase in adjusted *R*^2^ ranged from 5.9% to 24.2%, *p* < 0.001).

**Conclusions:** Two facets of mindfulness, “observing” and “acting with awareness,” were related to positive outcomes of psychotic patients after participating in the MBPEG. More efforts in addressing these two facets of mindfulness can be considered to increase the efficacy of mindfulness-based interventions in psychosis.

## Introduction

Mindfulness, with its roots in ancient Buddhist teaching, has been defined in a variety of ways so as to fit into the context of contemporary psychological science (1). Mindfulness is frequently described as a form of purposively and non-judgmentally paying attention (or being aware of) to unfolding moment-by-moment experiences with openness, curiosity, and acceptance (2–4). Practicing mindfulness is associated with a range of positive outcomes in diverse chronic and disabling conditions, including reduced stress and emotional distress, more positive thoughts and better quality of life (5–7). Mindfulness-based interventions, such as a combination of mindfulness training and psychoeducation program, have become increasingly popular in recent years, and they have been found to be more effective on improving psychological well-being, when compared to mindfulness and/or meditation training alone (8). There is a growing body of evidence from meta-analyses highlighting the significant effects of mindfulness-based interventions on psychological well-being and physical health outcomes of individuals diagnosed with cancer (9), chronic pain (10), psychiatric disorders (11), as well as healthy individuals (12). In a recent meta-analysis of ten randomized controlled trials (RCTs) of mindfulness- and acceptance-based interventions in adults with psychotic disorders, group format mindfulness-based interventions showed larger therapeutic effects (Hedge's *g* = 0.46, 95% CI: 0.18–0.75) than individual-based Acceptance and Commitment Therapy (*g* = 0.08, 95% CI: −0.23 to 0.38) (13).

While several trials have supported the effects of mindfulness-based interventions for psychotic patients (14–17), the mechanism that explains how mindfulness skills benefit psychotic patients has been underexplored. There is evidence that mindfulness-based interventions can improve mental health and well-being through reducing rumination and worry in adults with depressive symptoms, anxiety disorders and cancer (18). Other studies have also shown that changes in the levels of mindfulness in non-psychotic patients, such as those diagnosed with cancer or depression, are associated with perceived stress and other clinical outcomes, such as depression and anxiety (19, 20). However, it is currently not clear if increases in self-reported levels of different facets of mindfulness are associated with the changes in individual important clinical and psychosocial health outcomes of psychotic patients after undergoing mindfulness training and practices.

To investigate the mechanism by which the overall and/or components (facets) of mindfulness may produce benefits, there has been a growing interest in the assessment of mindfulness using validated self-reported questionnaires (21, 22). Among the commonly accepted self-reported measures, the Five Facet Mindfulness Questionnaire (FFMQ) (21) has been one of the most widely used and comprehensive measures of one's perceived levels of mindfulness in daily life (1). It consists of 39 items extracted and modified from five main mindfulness measures, including the Mindfulness Attention Awareness Scale (23), the Freiburg Mindfulness Inventory (24), the Kentucky of Mindfulness Skills (25), the Cognitive and Affective Mindfulness Scale—Revised (26), and the Southampton Mindfulness Questionnaire (27). The FFMQ has been validated (28) and found to include five facets of mindfulness, namely, “Observing” (noticing or attending to internal feelings and thoughts and external simulation); “Describing” (labeling feelings, thoughts and experiences with words); “Acting with awareness” (attending to what is happening in the present); “Non-judging of Inner Experience” (taking a non-evaluative stance toward internal thoughts and feelings); and “Non-reactivity to Inner Experience” (allowing emotions and thoughts to come and go, without being interfered by them). These five facets consolidate and reflect the five essential and most important aspects of mindfulness in current standardized approaches to mindfulness therapy, mainly Mindfulness-based Stress Reduction (MBSR) and Mindfulness-based Cognitive Therapy (MBCT).

Several previous studies on stress, depression and anxiety have examined the relationships between the overall and individual facets of mindfulness and a few health outcomes. Baer et al. (29) evaluated the effectiveness of an 8-week MBSR intervention and found that change in overall mindfulness skills during the first 3 weeks predicted change in perceived stress over the intervention period. In another study, an increase in mindfulness was found to fully mediate the relationships between meditation practices during the intervention and reductions in psychological symptoms and perceived stress (30). Cash and Whittingham (31) found that higher level of “non-judging” predicted lower levels of depression, anxiety and stress-related symptoms; whereas, higher level of “non-reactivity” predicted lower depressive symptoms. However, the aforementioned studies mainly targeted mood and stress-related conditions. There is a lack of investigation or understanding of the relationships between the changes in the five facets of mindfulness and other important health outcomes, such as psychosocial functioning, insight into illness/treatment and level of recovery in different severe mental illnesses.

In the context of contemporary psychological science, current methods of establishing construct validity of an instrument emphasize the importance of measuring each facet of a multifaceted construct via a unidimensional subscale (32). Hence, inconsistent with the Buddhist conceptions viewing mindfulness as a whole concept, mindfulness is often understood and measured in a multidimensional manner to cover different body-mind concepts, mainly including present-moment awareness, non-judging and non-reactivity (1). To better understand the nature and concepts of mindfulness and relationships between its dimensions/facets and level of patient functioning (1), it is interesting and important to know which facet(s) of mindfulness would show association(s) with individual health outcomes in psychotic patients. With this enhanced understanding, mindfulness-based interventions could be designed to address specific facets of mindfulness in order to maximize improvements in the targeted outcomes and optimize positive effects in specific patient groups.

This study aimed to examine whether and in what way the five facets of mindfulness were associated with the changes in the level of psychotic symptoms, subjective recovery, insight into illness/treatment, and functioning of patients with psychotic disorders after participating in a six-month mindfulness-based psychoeducation group (MBPEG) program. These relationships would be examined in consideration with the main socio-demographics and clinical characteristics of the patients. We expected that, in general, improvements in the five facets of mindfulness would be associated with improvements in the aforementioned health outcomes of these psychotic patients after participating in the MBPEG program over a 24-month follow-up.

## Materials and Methods

Longitudinal follow-up outpatient data from two RCTs conducted between 2013 and 2016 (ClinicalTrials.gov registration: NCT01667601) for psychotic patients during their early stage of illness (within 5 years of onset) were used for data analyses of this study (15, 17). These RCTs examining the effects of a mindfulness-based psychoeducation group (MBPEG) program for people with psychotic disorders over a 24-month follow-up period have been described in our earlier published articles (15, 17). In this paper, we report the within-group effects of the MBPEG program for psychotic patients on their perceived abilities in performing five facets of mindfulness by pooling the data from two treatment (MBPEG) groups. These treatment groups consist of one mindfulness-based intervention group from each of the two RCTs; and these results have not been previously reported. This analysis does not include data from participants randomly allocated into the conventional psychoeducation and treatment-as-usual (TAU) groups in the two RCTs because they did not receive mindfulness training and therefore the five facets of mindfulness were not measured.

### Participants

Patients were eligible for inclusion in the RCTs if they were aged between 18 and 64 years, diagnosed according to DSM-IV as psychosis (DSM-IV diagnostic code: 298.8, 297.1, 293.81, 293.82, or 297.3), schizophrenia, schizophreniform/schizoaffective disorders or other psychotic disorders (DSM-IV diagnostic code: 298.9) for <5 years by psychiatrists using the Structured Clinical Interview (33) as shown in their hospital records, and able to understand Mandarin/Cantonese. Participants were excluded if they were engaged in any mindfulness or other structured psychotherapeutic intervention(s) in the past year and diagnosed with co-morbidity of organic brain injury, learning disability or another mental disorder. A total of 124 patients (87%) who completed both the MBPEG program and 24-month follow-up assessments were included in the final data analysis. Sample size calculations were not conducted a-priori, but the sample size is considered as adequately powered for the two-block multiple regression analysis using the rule of thumb (*N* > 50 + 8 x number of independent variables) (34), hence being estimated to be not <114.

### Procedures

Briefly, from the two studies, we recruited a total of 449 randomly selected adult patients with psychotic disorders from nine outpatient clinics of three geographical regions (Taiwan, mainland China and Hong Kong). After completing the baseline assessments, the patients were randomly allocated in a 1:1:1 ratio to one of three intervention groups (mindfulness-based psychoeducation group, MBPEG; conventional psychoeducation group, CPEG; and treatment-as-usual, TAU). Each patient underwent six months of intervention, with the follow-up assessments conducted at 1-week and 6-, 12-, and 24-months post-intervention. The primary outcomes were the Positive and Negative Syndrome Scale (PANSS) score and duration of re-hospitalizations. Secondary outcomes included the Questionnaire about the Process of Recovery (QPR) score, Insight and Treatment Attitude Questionnaire (ITAQ) score and Specific Level of Functioning Scale (SLOF). Only those patients in the MBPEG group completed the Five Facet Mindfulness Questionnaire (FFMQ) and subsequently were included in this study.

The MBPEG program was fully described in our published articles (15, 17). In short, the program consisted of 12 biweekly sessions (24 weeks), with 10–12 patients participating in each two-hour group session. Based on Kabat-Zinn's MBSR, the content of the MBPEG was modified and validated for use in Chinese psychotic patients (17). The program consisted of three phases. Phase one was conducted over six sessions involving three components (“Orientation and engagement;” “Focused awareness on bodily sensations, thoughts, feelings and symptoms;” and “Empowering self-control of symptoms and negative thoughts”). Phase two (three sessions) consisted of two psychoeducational components: knowledge of schizophrenia and illness management/problem solving. The final phase (three sessions) involved behavioral rehearsals of relapse prevention and discussion about community resources/planning for the future. During all group sessions, the patients were taught and supervised to practice body scan and other mindfulness exercises for enhancing attention and awareness of breath, bodily sensations, thoughts, and emotions, as well as mindful sitting and/or walking. In addition, they were encouraged and reminded to practice mindfulness regularly (not <20 min twice daily) as homework assignments. At the later stage, the patients were also encouraged to cultivate an accepting attitude and positive thoughts/responses to life problems, and to develop a “decentered” attitude on their thoughts and feelings.

### Measures

Data collected at 0, 6, 12, and 24 months post-intervention were included in the analyses. Outcomes included the number of hospital admissions, the duration of hospital readmissions, the patients' symptoms (PANSS score), level of recovery perceived by patients (QPR score), insight into the illness/treatment (ITAQ score), level of functioning (SLOF score), and the five facets of mindfulness (FFMQ subscale scores).

#### Positive and Negative Syndrome Scale (PANSS)

The severity and impact of psychotic symptoms on behavior was assessed using the Positive and Negative Syndrome Scale (PANSS). The PANSS composes of 30 items across three subscales: positive symptoms (7 items, score range = 7–49), negative symptoms (7 items, score range = 7–49) and general psychopathology (16 items, score range = 16–112). Each item is rated from 1 (absent) to 7 (extreme). The total score ranges from 30 to 210. Higher scores indicate more severe symptoms. The PANSS has demonstrated good to excellent interrater reliability [intra-class correlation (ICC) = 0.88], internal consistency (Cronbach's alphas = 0.87–0.93) and concurrent and predictive validity (35).

#### Questionnaire About the Process of Recovery (QPR)

The patient's level of recovery was assessed using the Questionnaire about the Process of Recovery (QPR) (36). The QPR was previously translated and validated for use in Hong Kong Chinese patients (37). The QPR consists of 22 items for three subscales (10 items for Self-empowerment, 6 items for Effective interpersonal relationships and 6 items for Rebuilding life). Each item is self-rated on a five-point Likert scale (from “0 = disagree strongly” to “4 = agree strongly”). The total score ranges from 0 to 88. Higher total scores suggest higher levels of satisfaction about the level/progress of recovery. The Chinese version has demonstrated very satisfactory internal consistency (Cronbach's alphas = 0.88–0.90), sensitivity to different symptom severity groups (*t* = 5.34, *p* = 0.005) and test-retest reliability (ICC = 0.87–0.92) in Chinese patients with psychosis (37).

#### Insight and Treatment Attitude Questionnaire (ITAQ)

The Insight and Treatment Attitude Questionnaire (ITAQ) (38) is an 11-item scale measuring patients' awareness of illness (5 questions) and attitudes toward medication/hospitalization/follow-up (6 questions). Items are rated on three-point Likert scale, ranging from 0 = “Not necessary to receive medication/treatment” to 2 = “Medication/treatment should be continued/required regularly.” The total ITAQ scores (ranging from 0–33) can be categorized into three groups (poor insight = score of 0–7, fair insight = 8–14, good insight = 15 or above) (39). The ITAQ has been regularly used in patients with schizophrenia, and its validated Hong Kong Chinese version has shown good internal consistency (Cronbach's alphas = 0.85–0.88) and test-retest reliability(*r* = 0.80–0.86) (40).

#### Specific Level of Functioning Scale (SLOF)

Patient functioning was measured using the 43-item Specific Level of Functioning Scale (SLOF) (41). The SLOF items are self-rated on a five-point Likert scale (1 = poorest function to 5 = best function) and consist of three domains: physical functioning/personal care (12 items), social functioning (14 items), and community living skills (17 items). Higher scores suggest better functioning. The validated Chinese version has demonstrated satisfactory content validity, test-retest reliability (*r* = 0.76) and internal consistency (Cronbach's alphas = 0.88–0.96) in Hong Kong patients with schizophrenia (42).

#### Five Facet Mindfulness Questionnaire (FFMQ)

The FFMQ (21) is a 39-item self-completed questionnaire measuring the five facets of mindfulness: Observing (8 items), Describing (8 items), Acting with awareness (8 items), Non-judgmental (8 items), and Non-reactive (7 items). Participants rated the items on a five-point Likert scale (1 = never or very rarely true to 5 = very often or always true), each facet score ranges from 8 to 40, except for the non-reactive facet which ranges from 7 to 35. Higher scores indicate higher levels of mindfulness in terms of the scored facets. The Chinese version of the FFMQ has demonstrated acceptable internal consistency and test-retest reliability in Chinese people; and confirmatory factory factor analysis supported the five-factor model (28).

### Data Analyses

Normality of continuous variables was assessed by skewness statistics and normal probability plots. Data were summarized and presented using appropriate descriptive statistics. Area under the curve (AUC) of each repeated outcome measure and each facet of mindfulness measured at 0, 6, 12, and 24 months were computed using trapezoidal method. The AUC of each variable was then rescaled to the original possible range of the underlying variable and used for inferential analysis. Repeated-measures of analysis of variance test (repeated-measures ANOVA) was performed to examine any significant within-group change for each outcome measure and each mindfulness facet score over time. Correlations between AUCs of the five facets of mindfulness and other outcomes were assessed using Pearson correlation coefficients. Univariate analyses were conducted to examine the association between each patient socio-demographic/clinical characteristics and each outcome including symptom severity (PANSS total score), recovery (QPR total score), insights to illness and treatment (ITAQ total score), and psychosocial functioning (SLOF total score). Those characteristics that were significantly associated with the outcomes in the univariate analyses were subsequently included as covariates in the two-block multiple regression models with the five mindfulness facets as the predictors. Specifically, for each outcome, those socio-demographic and clinical characteristics with *p* < 0.05 in univariate analyses were all entered in the first block. Next, all the five facets of mindfulness variables were subjected to a backward mode in block 2 until only the facets of mindfulness variables with *p* < 0.05 were retained. All statistical analyses were performed using IBM SPSS 24 (IBM Crop., Armonk, NY). All statistical tests involved were two-sided with the level of significance set at 0.05.

## Results

From the two RCTs (15, 17), a total of 150 participants with psychiatric disorders were randomly allocated to the six-month MBPEG program. Of these, 36 were from Chien et al. (17) and 114 were from Chien et al. (15). Twenty-six participants (17.3%) dropped out or withdrew from the study over 24 months follow-up, including 6 in Chien et al. (17) and 20 in Chien et al. (15). Hence, a total of 124 participants were included in our analysis. Reasons for attritions were due to having lost contact during intervention (*n* = 15) or 1-week post-intervention (*n* = 4), withdrawal from the study/intervention due to loss of interest (*n* = 4) or absence from >4 intervention sessions (*n* = 3).

Table 1 presents the socio-demographic and clinical characteristics of the participants. Their mean age was 28.4 years (*SD* = 7), and 52% were male. The majority were single, divorced or widowed (59%), attained secondary school or below education (60%), had a full- or part-time job (83%), were living in public housing flats (51%) with their families or close friends (73%), and had received financial assistance (63%). More than half of the participants were diagnosed with recent-onset psychosis or other psychotic disorders (52%) and less than one third were diagnosed with schizophrenia (31%). The mean duration of illness, the mean duration of using anti-psychotics and the number of mental health services or therapies received were 15.9 months (*SD* = 5.5), 13.0 months (*SD* = 5.0), and 3.2 times (*SD* = 1.1), respectively. The majority of the participants (77%) had one or more chronic medical/physical illnesses such as hypertension, diabetic mellitus, renal disease, coronary heart disease, and other cardiovascular diseases. Most participants had taken moderate or high dosages of oral anti-psychotics (54%), had outpatient department use (95%), but had not used any day hospital or center services (76%) or substances recently (67%).

**Table 1:** Baseline characteristics of the participants (*N* = 124).

	***n***	**%**
Age, *M* (*SD*)	28.4 (7.0)	
Sex		
Male	65	52.4
Female	59	47.6
Marital status		
Single/divorced/widowed	73	59.0
Married/cohabit	51	41.0
Educational level		
Secondary school or below	74	59.7
College or university or above	50	40.3
Employment status		
Full-time employed	39	31.5
Part-time employed	64	51.6
Unemployed/others	21	16.9
Type of housing		
Public	63	50.8
Private	30	24.2
Supported accommodation/hostel/others	31	25.0
Living condition		
Living with family/close friends	90	72.6
Living alone	22	17.7
Residential/supervised	12	9.7
Received any financial assistance		
No	46	37.1
Yes	78	62.9
Diagnosis		
Psychosis	54^a^	43.5
Schizophrenia	38	30.7
Schizophreniform disorder	16	12.9
Schizoaffective disorder	5	4.0
Other psychotic disorders	11^b^	8.9
Duration of illness (months), *M* (*SD*)	15.9 (5.5)	
Other chronic illness(es)^c^		
No	29	23.4
Yes, one diagnosis	49	39.5
Yes, two diagnoses	46	37.1
Dosage of oral anti-psychotics^d^		
Low	57	46.0
Moderate	53	42.7
High	14	11.3
Duration of using anti-psychotics (months), *M* (*SD*)	13.0 (5.0)	
Number of mental health services/ therapies currently received, *M* (*SD*)	3.2 (1.1)	
Current use of services in outpatient psychiatric departments		
No	6	4.8
Yes	118	95.2
Current use of services in day hospitals/centers		
No	94	75.8
Yes	30	24.2
Current use of substance		
No	83	66.9
Yes	41	33.1

*M, mean; SD, standard deviation; n, number of subjects per category*.

a *Psychosis included: brief psychotic disorder (DSM-IV code: 298.8; n = 28); delusional disorder (DSM-IV code: 297.1; n = 8); psychotic disorder due to general medical conditions with delusions (DSM-IV code: 293.81; n = 7) and with hallucinations (DSM-IV code: 293.82; n = 9); shared psychotic disorder (297.3; n =2)*.

b *Other psychotic disorders (DSM-IV code: 298.9)*.

c *Other chronic illnesses included other chronic medical/physical illnesses, such as hypertension (n = 39), diabetic mellitus (n = 22), renal disease (n = 14), coronary heart disease (n = 10), and other cardiovascular diseases (n =24)*.

d *“High dose” referred to a ratio of prescribed daily dose (PDD) to defined daily dose (DDD) >1.5, doses over 1 g/day in chlorpromazine equivalent or dosage exceeding the recommended British National Formulary limits in case of multiple anti-psychotics used (43). “Low dose” referred to an average dose of ≥0.5 and <1 DDD unit or <0.5 g/day of chlorpromazine equivalent; and “moderate dose” was defined as an average dose of 1–1.5 DDD unit or 0.5–1.0 g/day of chlorpromazine equivalent (44)*.

Table 2 shows the levels of the symptom severity (PANSS total score), recovery (QPR total score), insights to illness and treatment (ITAQ total score), and psychosocial functioning (SLOF total score) as well as the FFMQ facet scores (Observing, Describing, Acting with Awareness, Non-Judging of Inner Experience, and Non-Reactivity to Inner Experience) across the four data collection time points (0, 6, 12, and 24 months). Furthermore, a summary measure based on area under the curve (AUC) of each variable measured over the 24-month period was computed using trapezoidal method to quantify the overall level of the variable of interest in that period. The AUC of each variable was then rescaled to the original possible range of the underlying variable for ease of comparison, which is also presented in Table 2. Repeated measures of ANOVA showed that all the scores showed significant linear trends across the time points at 0, 6, 12 and 24 months (all *p* < 0.001).

**Table 2:** Means, standard deviations, and possible ranges of outcomes and five facets of mindfulness across time.

**Mean (*SD*)**	**Possible range**	**Baseline**	**6-months**	**12-months**	**24-months**	**AUC^a^**
Outcomes						
PANSS total score	30–120	88.5 (9.3)	75.7 (10.4)	53.8 (13.7)	43.9 (14.7)	61.1 (11.6)
QPR total score	0–88	47.3 (3.9)	55.1 (3.9)	60.6 (3.6)	65.8 (3.8)	58.8 (3.3)
ITAQ total score	0–22	8.7 (3.0)	14.4 (2.6)	16.8 (1.7)	17.8 (1.6)	15.4 (1.5)
SLOF total score	43–215	121.3 (13.7)	143.5 (12.8)	161.8 (13.4)	182.6 (12.3)	157.4 (10.3)
Five facets of mindfulness						
FFMQ—Observing	8–40	12.5 (1.4)	16.0 (1.7)	18.9 (1.8)	23.1 (2.4)	18.4 (1.3)
FFMQ—Describing	8–40	12.6 (1.4)	16.1 (1.9)	19.3 (1.8)	24.1 (3.0)	18.8 (1.5)
FFMQ—Acting with awareness	8–40	11.9 (1.6)	15.3 (1.6)	18.6 (1.9)	23.2 (3.4)	18.1 (1.5)
FFMQ—Non-judging of inner experience	8–40	11.6 (1.6)	14.7 (1.8)	17.5 (1.6)	20.5 (2.4)	16.8 (1.4)
FFMQ—Non-reactivity to inner experience	7–35	12.1 (1.9)	15.8 (2.1)	17.5 (2.0)	20.6 (2.9)	17.2 (1.6)

*AUC, Area Under the Curve; p, p-value; SD, standard deviation; FFMQ, Five Facet Mindfulness Questionnaire; ITAQ, Insight and Treatment Attitude Questionnaire; PANSS, Positive and Negative Syndrome Scale; QPR, Questionnaire about the Process of Recovery; SLOF, Specific Level of Functioning Scale*.

a *The area under the curve of each variable spanned over the time from baseline to 24 months as estimated by trapezoidal method and rescaled to the original range of the underlying variable*.

The relationships between the five facets of mindfulness and other outcomes were assessed by the Pearson correlation coefficients between their AUC values (Table 3). The mindfulness facets were significantly correlated with other outcomes (all *p*-values ranged from <0.001 to <0.05), except the correlation between non-judging of inner experience and symptom severity (PANSS total score), which was insignificant (Table 3).

**Table 3:** Correlations between AUCs of the five facets of mindfulness and outcomes.

	**Observing**	**Describing**	**Awareness**	**Non-judging**	**Non-reactivity**
PANSS total score	−0.281^**^	−0.206^*^	−0.288^**^	−0.144	−0.295^***^
QPR total score	0.535^***^	0.509^***^	0.536^***^	0.282^**^	0.546^***^
ITAQ total score	0.607^***^	0.546^***^	0.536^***^	0.374^***^	0.537^***^
SLOF total score	0.554^***^	0.532^***^	0.516^***^	0.500^***^	0.356^***^

*AUC, Area Under the Curve from baseline to 24-months post-intervention; ITAQ, Insight and Treatment Attitude Questionnaire; PANSS, Positive and Negative Syndrome Scale; QPR, Questionnaire about the Process of Recovery; SLOF, Specific Level of Functioning Scale*.

* p < 0.05,

** p < 0.01,

*** *p < 0.001*.

Table 4 presents the univariate analyses between patient characteristics and outcomes. Almost all patient characteristics assessed in this study were significantly associated with at least one outcome (all *p*-values ranged from <0.001 to 0.043), except type of housing (all *p*-values ranged from 0.120 to 0.828), history of receiving financial assistance (all *p values* ranged from 0.190 to 0.832), current use of services in day hospitals/centers (all *p*-values ranged from 0.061 to 0.625). The variables with significant associations with each outcome (i.e., *p* < 0.05) were subsequently entered in a two-block multiple regression model for examining the associations between the five facets of mindfulness and the outcome of interest.

**Table 4:** Univariate association analyses between patient characteristics and outcomes.

	**PANSS total score^a^**		**QPR total score^a^**		**ITAQ total score^a^**		**SLOF total score^a^**	
**Characteristics**	**Mean (*SD*)/*r***	***p***	**Mean (*SD*)/*r***	***p***	**Mean (*SD*)/*r***	***p***	**Mean (*SD*)/*r***	***p***
Age (years)	0.105^b^	0.246	−0.401^b^	<0.001	−0.443^b^	<0.001	−0.308^b^	<0.001
Sex								
Male	61.4 (12.2)	0.784	59.1 (3.1)	0.410	15.6 (1.4)	0.117	159.3 (10.0)	0.032
Female	60.8 (11.1)		58.6 (3.6)		15.2 (1.7)		155.3 (10.4)	
Marital status								
Single /divorced/widowed	59.1 (11.6)	0.031	59.7 (3.2)	<0.001	15.8 (1.4)	<0.001	158.8 (9.2)	0.037
Married/cohabit	63.7 (11.4)		57.5 (3.1)		14.8 (1.6)		154.8 (11.3)	
Educational level								
Secondary school or below	61.3 (12.6)	0.871	58.4 (2.7)	0.092	15.1 (1.4)	0.017	155.3 (9.8)	0.007
College or university or above	60.9 (10.2)		59.5 (4.0)		15.8 (1.6)		160.4 (10.5)	
Employment status								
Full-time employed	62.5 (11.0)	0.007	59.9 (3.6)	0.001	16.0 (1.6)	0.012	159.2 (9.9)	0.383
Part-time employed	62.9 (9.7)		57.9 (3.2)		15.0 (1.6)		156.6 (11.1)	
Unemployed/others	53.0 (14.8)		59.8 (2.2)		15.5 (1.0)		156.1 (8.5)	
Type of housing								
Public	61.3 (11.5)	0.120	58.7 (3.4)	0.828	15.2 (1.6)	0.520	156.3 (11.1)	0.448
Private	64.1 (7.9)		58.8 (3.1)		15.6 (1.1)		158.2 (5.6)	
Supported accommodation/ hostel/other	57.9 (14.3)		59.2 (3.4)		15.5 (1.7)		158.8 (12.1)	
Living condition								
Living with family/close friends	61.6 (9.5)	<0.001	59.0 (3.4)	0.140	15.5 (1.5)	0.393	158.7 (8.8)	0.048
Living alone	68.2 (12.2)		57.8 (3.7)		15.0 (2.0)		154.3 (14.9)	
Residential/ supervised	44.4 (9.7)		59.6 (1.4)		15.5 (1.2)		152.7 (9.5)	
Received any financial assistance								
No	59.3 (10.8)	0.190	58.8 (3.4)	0.832	15.3 (1.5)	0.656	158.4 (9.4)	0.403
Yes	62.2 (12.0)		58.9 (3.3)		15.5 (1.6)		156.8 (10.9)	
Diagnosis								
Schizophrenia	60.4 (10.6)	0.013	59.2 (3.7)	0.058	15.6 (1.7)	0.061	158.6 (10.1)	0.178
Psychotic disorders	58.7 (15.2)		58.0 (2.5)		14.9 (1.2)		155.3 (11.4)	
Schizophreniform disorder/Schizoaffective disorder^c^	69.2 (5.7)		58.2 (1.9)		15.4 (1.0)		154.4 (9.1)	
Duration of illness (months)	−0.245^b^	0.006	−0.134^b^	0.139	−0.209^b^	0.020	−0.355^b^	<0.001
Other chronic illness(es)								
No	69.3 (10.6)	<0.001	57.6 (3.3)	0.043	15.2 (1.9)	0.190	158.5 (13.9)	0.101
Yes, one diagnosis	60.7 (8.3)		59.6 (3.0)		15.7 (1.3)		159.1 (9.1)	
Yes, two diagnoses	56.5 (12.7)		58.8 (3.5)		15.2 (1.6)		154.8 (8.5)	
Dosage of oral anti-psychotics								
Low	61.7 (12.4)	0.635	58.5 (3.5)	<0.001	15.2 (1.7)	<0.001	155.3 (12.6)	<0.001
Moderate	61.1 (12.3)		58.2 (2.7)		15.2 (1.2)		157.2 (7.3)	
High	59.0 (3.7)		62.6 (2.4)		17.2 (0.6)		166.6 (2.8)	
Duration of using anti-psychotics (months)	−0.197^b^	0.028	−0.078^b^	0.391	−0.135^b^	0.135	−0.268^b^	0.003
Number of mental health services/ therapies currently received	−0.067^b^	0.458	−0.109^b^	0.229	−0.111^b^	0.218	−0.107^b^	0.235
Current use of services in outpatient psychiatric departments								
No	69.2 (1.8)	<0.001	61.1 (2.2)	0.083	16.3 (0.7)	0.131	166.2 (6.0)	0.031
Yes	60.7 (11.8)		58.7 (3.3)		15.4 (1.6)		156.9 (10.3)	
Current use of services in day hospitals/centers								
No	60.0 (11.7)	0.061	58.7 (3.3)	0.497	15.4 (1.5)	0.625	156.9 (9.5)	0.361
Yes	64.6 (11.0)		59.2 (3.5)		15.5 (1.6)		158.9 (12.7)	
Current use of substance								
No	62.3 (8.3)	0.196	59.2 (3.6)	0.057	15.7 (1.6)	0.004	160.3 (8.7)	<0.001
Yes	58.7 (16.3)		58.1 (2.5)		14.9 (1.3)		151.4 (10.9)	

*AUC, Area Under the Curve; SD, standard deviation; p, p-value; r, Pearson correlation coefficient; ITAQ, Insight and Treatment Attitude Questionnaire; PANSS, Positive and Negative Syndrome Scale; QPR, Questionnaire about the Process of Recovery; SLOF, Specific Level of Functioning Scale*.

a *The area under the curve of each outcome spanned over the time from baseline to 24 months was used as dependent variable*.

b *Pearson correlation coefficient*.

c *Since the frequency of schizoaffective disorder is too small (n = 5), it was combined with the frequency of schizophreniform disorder (n = 16)*.

As shown in Table 5, variables related to patient characteristics accounted for at least 40% of variability of outcomes in block 1 (*R*^2^ ranged from 0.40 to 0.50). The backward regression analyses in block 2 revealed that among the five facets of mindfulness, only “observing” was significantly associated with symptom severity (PANSS total score) (adjusted *R*^2^ increase = 5.9%, *p* < 0.001). Likewise, the regression analysis with recovery (QPR total score) as the outcome found only “acting with awareness” was a significant predictor (adjusted *R*^2^ increase = 16.4%, *p* < 0.001). Two facets of mindfulness, “observing” and “acting with awareness,” were significantly associated with insight into illness and treatment (ITAQ total score) (adjusted *R*^2^ increase = 19.1%, *p* < 0.001), while only “observing” was significantly associated with psychosocial functioning (SLOF total score) (adjusted *R*^2^ increase = 24.2%, *p* < 0.001).

**Table 5:** Regression analyses to identify the facets of mindfulness independently and significantly associated with outcomes.

	**PANSS total score^a^^,^^b^**			**QPR total score^a^^,^^c^**			**ITAQ total score^a^^,^^d^**			**SLOF total score^a^^,^^e^**		
**Independent variables**	**β**	***SE***	***p***	**β**	***SE***	***p***	**β**	***SE***	***p***	**β**	***SE***	***P***
Block 1												
Patient characteristics significantly associated with each underlying outcome in the univariate analyses as shown in Table 4 were entered	*R*^2^ = 0.499			*R*^2^ = 0.455			*R*^2^ = 0.431			*R*^2^ = 0.400		
Block 2^f^												
Observing	−2.547	0.673	<.001	–	–	–	0.316	0.125	0.013	4.960	0.581	<.001
Describing	–	–	–	–	–	–	–	–	–	–	–	–
Acting with awareness	–	–	–	1.114	0.161	0.001	0.339	0.117	0.004	–	–	–
Non-judging of inner experience	–	–	–	–	–	–	–	–	–	–	–	–
Non-reactivity to inner experience	–	–	–	–	–	–	–	–	–	–	–	–
	Total *R*^2^ = 0.557 (Δ*R*^2^ = 0.058, *p* < 0.001)			Total *R*^2^ = 0.619 (Δ*R*^2^ = 0.164, *p* < 0.001)			Total *R*^2^ = 0.622 (Δ*R*^2^ = 0.191, *p* < 0.001)			Total *R*^2^ = 0.642 (Δ*R*^2^ = 0.242, *p* < 0.001)		

*β, unstandardized beta-coefficient; SE, standard error; p, p-value; R^2^, Multiple correlation squared; –, not retained in backward selection of the five independent variables; AUC, Area Under the Curve; ITAQ, Insight and Treatment Attitude Questionnaire; PANSS, Positive and Negative Syndrome Scale; QPR, Questionnaire about the Process of Recovery; SLOF, Specific Level of Functioning Scale*.

a *The area under the curve of each outcome spanned over the time from baseline to 24 months was used as dependent variable*.

b *Marital status, employment status, living condition, diagnosis, duration of illness, number of other chronic illness(es), duration of using anti-psychotics, and use of services in outpatient psychiatric departments were included as covariates for the regression analysis*.

c *Age, marital status, employment status, number of other chronic illness(es), and dosage of oral anti-psychotics were included as covariates for the regression analysis*.

d *Age, marital status, educational level, employment status, duration of illness(es), dosage of oral anti-psychotics, and current use of substances were included as covariates for the regression analysis*.

e *Age, sex, marital status, educational level, living condition, duration of illness, dosage of oral anti-psychotics, duration of using anti-psychotics, outpatient department use, and current use of substance were included as covariates for the regression analysis*.

f *Backward selection of the five facets of mindfulness that significantly associated with each underlying outcome in Block 2*.

## Discussion

The present study aimed to examine the role of mindfulness in improving the health outcomes of psychotic patients who participated in a six-month mindfulness-based psychoeducation (MBPEG) program by using the data from our RCTs conducted in Taiwan, Hong Kong and Mainland China (15, 17). In order to achieve this aim we report the effects of the MBPEG program on the five facets of mindfulness, as well as the relationships between the changes of the five facets and the psychological health outcomes of psychotic patients.

There was a significant within-group effect of the MBPEG program on the five facets of mindfulness starting from post-intervention to 24-month post-intervention. This is in line with the current evidence from three meta-analyses showing that the scores of self-reported mindfulness measures can noticeably increase in response to mindfulness training received (12, 19, 45). In addition, the correlation matrix in this study showed that the positive changes in the five facets of mindfulness were significantly associated with the positive changes in most of the patient outcomes. This result is consistent with the findings of a recent systematic review, indicating the increased mindfulness after participating in mindfulness-based interventions can mediate or be associated with improvements of several psychological health outcomes in people with anxiety disorders and depression (46). The improvements of the five facets of mindfulness and psychotic symptoms following the MBPEG could be related to the therapeutic effects of the intervention on reducing psychotic symptoms through the enhanced mindfulness, particularly at the later stage, or vice versa. Another possible explanation could be that after participating in the MBPEG program, the psychotic patients could learn how to relate their aversive experiences (i.e., internal and external stimuli, such as distressing thoughts, feelings and bodily sensations) differently (19). Hence, there could be a chance that the psychotic patients acquired better mindfulness skills and embedded mindfulness practices into their daily activities over the 24-month follow-up, leading to better self-management of the illness and improved psychotic symptoms. Nevertheless, these proposed inter-relationships between the five facets of mindfulness, symptom severity and important health outcomes of these psychotic patients, the directions of their changes, and the underlying reasons or mechanisms, deserve further research.

Among all four patient outcomes, psychosocial functioning was found to be the most responsive to the changes of the facets of mindfulness across time, as evidenced by the largest change of variance. Whereas psychotic symptoms were found to be the least responsive, with the smallest change of variance. These findings may highlight that although the general premise of mindfulness was delivered in the MBPEG program across all our trials, the specific mindfulness-based interventions were designed to improve the functional impacts of psychotic experiences, rather than to focus on reducing psychotic symptomology (47).

Patterns of the findings from the regression analyses show that among the five facets of mindfulness skills, only “observing” and “acting with awareness” are significantly associated with the improvements in health outcomes of psychotic patients after participating in the MBPEG program. Specifically, “observing” appeared to be the most influential facet, as it was associated with the improvements of three patient outcomes, including symptom reduction, insights into the illness and psychosocial functioning, after controlling for their clinical characteristics. Furthermore, “observing” was the only facet of mindfulness, which contributed the largest variance change in improved psychosocial functioning over 24-month post-intervention. In this study, the significant role of observing in improving patients' health outcomes could be due to the patients' increased ability of noticing or attending to present-moment experiences, which is a fundamental step for practicing mindfulness (48). Such practices may gradually increase attentional and behavioral control, ultimately leading to disruption of maladaptive thoughts and negative sensations (49). Indeed, a number of earlier studies have identified that an increase in “observing” (facet) is associated with or mediates the effects of mindfulness-based interventions in patients with post-traumatic stress disorders (50), diabetes (51), non-clinical samples with paranoia (52), depression and anxiety disorders (53), as well as psychological distress of breast-feeding mothers (54). However, it is still unclear if “observing” is the essential facet of mindfulness responsible for positive improvements in health outcomes across different conditions.

Although positive health outcomes have been found in psychotic patients after receiving mindfulness training (15, 17), there has been debate in the field that mindfulness may actually exacerbate symptoms of psychosis. This exacerbation is proposed to result from promoting awareness of internal states and other meditation-related adverse effects, such as meditation-induced psychosis, depersonalization, and mania (55). Of note, this evidence was mainly based on spontaneous reporting, case studies or observational studies, often in the absence of a control group (55). In line with the principle of mindfulness as suggested by Chadwick (2009), the MBPEG in our studies focused on “decentered awareness” in which psychotic patients could discover that much of their distress came from how they *reacted* to their psychotic experiences (distressing voices, images and thoughts), while mindfully observing and non-judgmentally accepting them could be alternative ways of *response*. Hence, as the patients' insight into their illness increased, they were more motivated to control their psychotic symptoms and became actively engaged with their treatment plans. In our studies (15, 17), the management strategies for potential risks were limited to excluding participants who were mentally unstable and those unable to provide informed consent, and no adverse events were found. Future research on the nature and scope of potential adverse events of mindfulness practice in psychotic patients should be considered in order to minimize potential risks and ascertain if specific types of patients are more susceptible to adverse effects.

### Study Strengths, Limitations, and Recommendations for Future Research

This study has several methodological strengths, such as the size of combined samples from two studies, as well as inclusion of relatively long-term follow-up data. Nevertheless, a few limitations of this study should be noted. First, given that the patients who were randomly allocated to the conventional psychoeducation program or TAU only in our RCTs did not receive mindfulness training, their five facets of mindfulness were not measured. Therefore, we did not include these patients' data in the analysis. Although the findings of the RCTs had already proved that the significant changes in the patient outcomes were mainly contributed to, or explained by, the effects of the MBPEG program, the combined or synergic effects of the mindfulness training/practices and psychoeducation could not be identified and differentiated from the mindfulness training alone. In this report, we only conducted within-group analyses to examine the associations between the changes in the five facets of mindfulness and the health outcomes found to have significantly improved across time. We were unable to follow the criteria as suggested by Kazdin (56, 57) to assess whether changes in the facets of mindfulness mediated the effects of the MBPEG program on the patient outcomes, when compared with the conventional psychoeducation or TAU alone. As suggested by Baer (1), participants who did not receive explicit mindfulness training could report with “mild to moderate increases in mindfulness score” because people might sometimes self-cultivate some related skills such as awareness and willingness to experience thoughts and feelings. It is also possible that the facets of mindfulness could change under other cognitive and behavioral interventions. For future studies assessing treatment effects of a mindfulness-based intervention and exploring the mechanism of actions/changes, the comparison group(s) should also include the assessment of the levels of mindfulness.

Second, it could be possible that although the MBPEG program improved patient outcomes, the improvements may have occurred through a mechanism other than increased mindfulness *per se*. For example, psychotic patients who had received MBPEG training may also be motivated to change or improve other health-related behaviors such as adherence to anti-psychotics or psychiatric outpatient service. In addition, apart from mindfulness, decentering, rumination, worry, and self-compassion have been recently suggested as the mediators leading to improvements of mental health outcomes (46). Future research should attempt to measure these variables, so that triangulation of measures and sufficient study power can be provided to elicit more explanatory information about the underexplored mechanism of mindfulness-based interventions.

Third, the fidelity to mindfulness practices in both RCTs was assessed with a checklist as suggested by the NIH Behavior Change Consortium recommendations covering the following five components: design, training, delivery, receipt, and enactment (58). Nevertheless, we were unable to evaluate whether all facets of mindfulness were delivered equally in the MBPEG or some of the facets were delivered more than the others.

Lastly, while the 39-item FFMQ used in this study has demonstrated very satisfactory test-retest reliability and construct validity (59), there is a debate in literature about the insufficient construct validity in self-reporting measures of mindfulness (55). Indeed, there is a widespread acknowledgment that information based on self-reported measures of complex psychological characteristics, such as mindfulness, cognitions and emotions, are inadequate (60). The use of a multimodal approach, where neurobiological and behavioral assessments can be used to capture attentional capacity, may be helpful to mutually inform mindfulness and establish process models of mindfulness for future research (55).

## Conclusion

The present study adds to existing research into mindfulness-based interventions for psychotic patients. We have shown that changes in two facets of mindfulness, “observing” and “acting with awareness,” appear to have important roles in improving psychotic symptoms, progress of recovery, insight into illness/treatment, and psychosocial functioning in patients with psychotic disorders.

### Study Implications

To date, mindfulness-based interventions usually adopt a range of distinct meditation techniques, such as body scan, walking meditation, and mindful breathing. However, to develop an effective intervention in a controlled trial, one logical step is to identify what specific facets of mindfulness are targeted by these meditation techniques, and then include the techniques that are the most effective on patient outcomes as the core components of the intervention. For example, body scan exercise is the most effective at cultivating “observing” and “observing” is the facet of mindfulness that was most strongly associated with positive health outcomes in psychotic patients. Therefore, focusing specifically on this exercise may improve the effectiveness of the mindfulness-based intervention. Considering cognitive changes in psychotic patients, more structured and focused approaches to mindfulness-based intervention, for instance, putting more efforts targeting at “observing” and “acting with awareness,” may allow these patients to achieve better functioning and illness insight. Our findings set the foundation for future research on designing a tailored mindfulness-based intervention for psychotic patients and exploring the mechanism of changes in the five facets of mindfulness, thus potentially resulting in better health outcomes of these patients.

## Data Availability Statement

The datasets generated for this study are available on request to the corresponding author.

## Ethics Statement

The study was granted ethical approvals by the Hospital Authority or Health Ministry governing the study clinics, and The University in Hong Kong (HSEARS20140218003). All study procedures complied with the ethical standards of the relevant institutional committees on human experimentation and the ethical standards laid down in the 1964 Declaration of Helsinki, and its later amendments. Written informed consent was obtained from all individual participants who were included in the study.

## Author Contributions

WC and DB: study design. WC: funding acquisition and study coordination and implementation. WC, KChow, YC, KChoi, and CC: data collection and analysis. WC, YC, KChoi, DB, and CC: drafting the manuscript. All authors approved the final version of the manuscript for submission.

### Conflict of Interest

The authors declare that the research was conducted in the absence of any commercial or financial relationships that could be construed as a potential conflict of interest.
